# The Impact of Intraoperative Hypothermia on Blood Loss and Allogenic Blood Transfusion in Total Knee and Hip Arthroplasty: A Retrospective Study

**DOI:** 10.1155/2020/1096743

**Published:** 2020-05-03

**Authors:** Pin Pan, Kai Song, Yao Yao, Tao Jiang, Qing Jiang

**Affiliations:** ^1^Department of Sports Medicine and Adult Reconstructive Surgery, Drum Tower Hospital, School of Medicine, Nanjing University, 321 Zhongshan Road, Nanjing, 210008 Jiangsu, China; ^2^Laboratory for Bone and Joint Disease, Model Animal Research Center (MARC), Nanjing University, Nanjing, 210093 Jiangsu, China

## Abstract

**Background:**

Total joint arthroplasty (TJA) usually leads to substantial blood loss, which may cause allogenic blood transfusion. Hypothermia occurring during operation has been reported to increase blood loss and transfusion rates in nonorthopedic cohorts. However, the relationship between intraoperative hypothermia and blood loss remains controversial in patients undergoing orthopedic surgeries. The aims of this study were to investigate the incidence of hypothermia and identify the impact of intraoperative body temperature and hypothermia on blood loss and transfusion rates in total knee and hip arthroplasty (TKA and THA, respectively).

**Methods:**

This retrospective study enrolled 616 consecutive patients, who underwent primary unilateral TKA or THA at our institution during the period from April 2012 to July 2014. The occurrence of a temperature below 36°C during the operation was documented to identify the incidence of hypothermia. Univariate analysis was performed to find the risk factors for hypothermia. Multiple regression analysis and multivariate logistic regression analysis were employed to explore the association of intraoperative temperature and hypothermia with intraoperative blood loss and perioperative blood transfusion.

**Results:**

The incidence of intraoperative hypothermia was 13.5%, 14.0%, and 13.1% in TJA, TKA, and THA, respectively. Intraoperative temperature (*P* = 0.045, *P* = 0.006) and hypothermia (*P* = 0.042, *P* < 0.001) were associated with intraoperative blood loss and perioperative transfusion in TKA. Intraoperative temperature (*P* = 0.002) was negatively related to the amount of blood loss, and hypothermia (*P* = 0.031) was the independent risk factor for transfusion in THA.

**Conclusion:**

Intraoperative hypothermia is associated with increased blood loss and transfusion rates in TJA. Efforts should be made to maintain normothermia during operation in these patients.

## 1. Introduction

Total joint arthroplasty (TJA) can cause substantial blood loss, which may lead to the requirement of allogeneic blood transfusion. Transfusion is associated with potential risks including transmission of infectious diseases, immunological reaction, cardiopulmonary complications, and increased length of stay [1, 2]. Therefore, the value of identifying modifiable risk factors for blood loss and transfusion is well recognized.

Intraoperative hypothermia has proven to be associated with higher rates of blood transfusion in nonorthopedic cohorts [3]. The relationship between intraoperative hypothermia and blood loss remains controversial in patients undergoing orthopedic surgeries [4]. Maintaining body temperature during spinal surgeries under general anesthesia reduces blood loss and transfusion rate. However, other studies did not find the association of hypothermia with blood loss in shoulder arthroplasty [5], pediatric posterior spinal fusion [6], and open lumbar spine surgery [7]. Hypothermia, which is defined as a core body temperature below 36°C [8], is a common event during operations due to the impacts of anesthetics and prolonged skin exposure to the cool environment of the operation room. It is also related to thermal discomfort, increased risk of cardiac morbidity, higher rates of surgical site infections, prolonged effects of anesthetics, and increased length of hospital stay [3, 9, 10].

Patients undergoing TJA are at high risk of hypothermia during surgery, especially in the setting of general anesthesia. Previous studies reported hypothermia rates of 11.2%-34% for total knee arthroplasty (TKA) and 13.2%-43.9% for total hip arthroplasty (THA) [11–13]. Hypothermia has proven to be associated with increased rates of readmission and deep infection [11]. However, whether hypothermia is related to increased blood loss and higher transfusion rates remains controversial in TJA [13, 14].

Therefore, we conducted this study in TKA and THA cohorts to determine the prevalence of intraoperative hypothermia and to explore the impact of intraoperative temperature and hypothermia on blood loss and transfusion rates.

## 2. Material and Methods

This study was approved by the Institutional Review Board (reference number: 2016-063-02). Patients who underwent primary unilateral TKA or THA consecutively at our institution between April 2012 and July 2014 were included in this retrospective study. If patients received staged TJA during the research period, only the first procedure was included. We excluded patients with coagulation disorders or on anticoagulants before surgery. Patient demographic and clinical data was collected from medical records, including age, gender, body mass index (BMI), preexisting illnesses (diabetes, hypertension, and malignancy), primary diagnosis, and operation time. The data of preoperative laboratory examinations, such as hemoglobin (Hb) concentration, blood platelet count (PLT), plasma prothrombin time (PT), international normalized ratio (INR), and activated partial thromboplastin time (APTT), was also documented.

Surgeries were performed by four experienced surgeons under general anesthesia. A tourniquet was used in all patients undergoing TKA. The tourniquet was inflated before skin incision and deflated after cementing the prosthesis. All patients received propofol-based total intravenous anesthesia. Continuous infusion of propofol, remifentanil, and cisatracurium was employed during the surgery. Ventilation was controlled to maintain end-tidal CO_2_ at 30-35 mmHg. All patients had normal body temperature before anesthesia. Active warming devices were not routinely used. If a temperature lower than 36°C was detected during the surgery, forced air warming would be used to maintain the body temperature. Body temperature was recorded via esophageal probe every 5 minutes during the surgery. In this study, the term “intraoperative temperature” used in the analysis was calculated by taking the average of temperature readings during the surgery, and the term “hypothermia” described a body temperature nadir lower than 36°C during the surgery. Intraoperative blood loss was calculated by the volume of blood in the suction canister plus the estimated amount of blood in the surgical sponges. The amount of blood in the suction canister was estimated by the total volume of fluid in the canister minus the volume of irrigation solution during the surgery. The surgical sponges were weighed before and after surgical use. The estimated amount of blood in the sponges was determined by calculating the weight difference before and after use. None of the patients received transfusion preoperatively. During the operation, blood transfusion was performed if the level of hemoglobin (Hb) decreased lower than 9.0 g/dl. After the surgery, patients with a hemoglobin level of less than 8.0 g/dl or developing symptomatic anemia with a hemoglobin level of less than 9.0 g/dl would receive transfusion. The events of perioperative transfusion were recorded. Drainage tubes were routinely placed in these patients and removed within 48 hours postoperatively. All patients received recommended thromboembolism prophylaxis regimen (rivaroxaban or low-molecular-weight heparin) after surgery. Tranexamic acid was not used routinely in these patients perioperatively.

Statistical analysis was conducted using Stata, version 12.0 (StataCorp LP, College Station, TX). To explore the risk factors for intraoperative hypothermia, we compared age, gender, BMI, preexisting illnesses, primary diagnosis, operation time, laboratory data, intraoperative blood loss, and perioperative blood transfusion between the hypothermia group and the nonhypothermia group. Qualitative variables were analyzed using a chi-square test, and quantitative variables were analyzed using a *t*-test. Multiple regression analysis was employed to determine whether intraoperative temperature and hypothermia are associated with blood loss after adjustment for age, gender, BMI, preexisting illnesses, diagnosis, preoperative laboratory data, and operation time. Similarly, we performed multivariate logistic regression analysis to explore whether intraoperative temperature and hypothermia affect perioperative transfusion rates independently.

## 3. Results

A total of 616 patients were enrolled in this study with a mean age of 65.9 ± 11.8 years (range 20-93 years). Two hundred and seventy-two patients received TKA, and 344 patients underwent THA. There were 426 females and 190 males. The mean intraoperative temperature was 36.4 ± 0.2°C (range 35.8-36.8°C). Eighty-three patients (13.5%) experienced an event of hypothermia during the surgery. The mean amount of intraoperative blood loss was 208.7 ± 131.3 ml (range 40-1050 ml). There were 94 patients (15.3%) receiving allogeneic transfusion during the perioperative period. The characteristics of these patients are summarized in Table 1.

There were 222 females and 50 males undergoing TKA with a mean age of 68.0 ± 7.3 years (range 40-86 years). They were diagnosed with osteoarthritis or rheumatoid arthritis. The mean intraoperative temperature was 36.4 ± 0.2°C (range 35.8-36.8°C). The incidence of intraoperative hypothermia was 14.0% (*n* = 38). The mean amount of intraoperative blood loss was 196.0 ± 103.2 ml (range 50-600 ml). Forty-eight patients (17.6%) received perioperative transfusion. According to the univariate analysis, we determined the association of intraoperative blood loss (*P* = 0.005) and perioperative transfusion (*P* < 0.001) with hypothermia in the TKA group (Table 2). Both intraoperative temperature (*P* = 0.045) and hypothermia (*P* = 0.042) were associated with intraoperative blood loss after adjustment using multiple regression analysis (Table 3). Subsequently, we conducted multivariate logistic regression analysis and found that intraoperative temperature (*P* = 0.006, OR = 0.080) and hypothermia (*P* < 0.001, OR = 4.468) were independently related to transfusion rates (Table 3).

A total of 344 patients received THA for femoral neck fracture, osteonecrosis of the femoral head, primary osteoarthritis, developmental dysplasia of the hip, rheumatoid arthritis, or ankylosing spondylitis. There were 204 females and 140 males with a mean age of 64.2 ± 14.1 years (range 20-93 years). The mean intraoperative temperature was 36.3 ± 0.1°C (range 36.0-36.7°C). Forty-five patients (13.1%) experienced an event of hypothermia intraoperatively. The mean amount of intraoperative blood loss was 218.7 ± 149.3 ml (range 40-1050 ml). The incidence of perioperative transfusion was 13.4% (*n* = 46). According to the univariate analysis, we determined the relationship between transfusion (*P* = 0.019) and hypothermia in the THA group (Table 4). The intraoperative temperature was negatively related to blood loss (*P* = 0.002) after adjustment in the multiple regression analysis. However, we did not find the relationship between the event of hypothermia and blood loss (*P* = 0.152) (Table 5). The multivariate logistic regression analysis indicated that hypothermia (*P* = 0.031, OR = 2.615) rather than intraoperative temperature (*P* = 0.073, OR = 0.122) was the independent risk factor for transfusion (Table 5). In the THA group, the hip fracture patients are different from the other patients in pathogenesis and clinical manifestation, which may influence the analysis. Therefore, we performed additional multivariate analysis without hip fracture patients. In these patients, we only found the negative relationship between intraoperative temperature and blood loss (*P* = 0.017) (data not shown).

## 4. Discussion

Inadvertent hypothermia occurs commonly during surgeries mainly due to skin exposure to cool environment, tissue loss, respiration, and disrupted thermoregulatory control caused by general anesthesia [15]. In this study, we identified the incidence of intraoperative hypothermia in TJA and attempted to explore the risk factors. The rates of intraoperative hypothermia were 13.5%, 14.0%, and 13.1% in TJA, TKA and THA, respectively. The incidence of hypothermia reported by previous studies varied widely from 11.2% to 43.9% [11–13], which may be caused by different warming methods, anesthesia procedure, and operating room environment. According to previous studies, female gender, lower BMI, knee arthroplasty, neuraxial anesthesia, lower preoperative temperature, lower operating room temperature, and longer operation time are the risk factors for hypothermia in TJA [11, 13, 16]. Therefore, measures improving the control of modifiable risk factors may be able to reduce the rate of hypothermia.

By employing multiple regression analysis and multivariate logistic regression analysis, intraoperative temperature and hypothermia were found to be independently associated with intraoperative blood loss and perioperative transfusion in TKA. In THA, intraoperative temperature was negatively related to the amount of blood loss, and hypothermia was the independent risk factor for transfusion. Rajagopalan et al. conducted a systematic review to evaluate the effects of hypothermia on surgical patients and found that even mild hypothermia increased blood loss and risk for transfusion [17]. Schmied et al. found that mild hypothermia increased transfusion requirements and augmented blood loss by approximately 500 ml in patients undergoing THA [18]. Frisch et al. investigated the impact of intraoperative hypothermia in TKA and THA and also found that hypothermia was associated with increased blood loss. However, the relationship between hypothermia and blood transfusion was not observed in their study [13]. The impaired coagulation system induced by hypothermia may explain the increased blood loss. Hypothermia has been proven to impair the coagulation cascade by inhibiting enzymatic reactions, which causes prolonged prothrombin time and partial thromboplastin time [19]. Mild hypothermia also reduces the adhesion ability of the platelet and the subsequent coagulation process [20]. Besides, hypothermia has been demonstrated to inhibit platelet activation in vitro and in vivo, which slows down the formation of a fibrin-rich hemostatic plug [21].

Intraoperative hypothermia also increases the risk of reoperation and postoperative infection in TJA [11]. Therefore, maintenance of normothermia may decrease the risk of postoperative complications and improve outcomes. Warming strategies are classified into passive and active warming systems. Passive warming systems refer to the layers of insulation, such as blankets and surgical drapes. Active warming systems include forced air, circulating water, radiant warmer, fluid warming, and airway heating [15]. For patients undergoing surgeries, the factor that contributes most to perioperative hypothermia is the internal redistribution of body heat caused by general or neuraxial anesthesia [22]. Therefore, it is important to monitor temperature and maintain normothermia in this population. Another reason for inadvertent hypothermia is the exposure of the skin to the environment of the operation room. Therefore, efforts should be made to maintain an optimal operation room temperature, which can reduce the risk of complications [23]. Body temperature has been reported to drop obviously between preoperative holding and induction of anesthesia [12]. A previous study used preoperative forced-air warming and found that it could significantly decrease the rate of intraoperative hypothermia [24].

There are several limitations in this study. First, the present study has all the inherent limitations of a retrospective study. Second, the “intraoperative temperature,” which we used for analysis, was calculated by taking the average of temperature readings from an esophageal detector. Since the readings were recorded at intervals of 5 minutes, the “intraoperative temperature” could not completely represent the temperature during the entire process of surgery. Third, the amount of intraoperative blood loss was estimated by the volume of blood in the suction canister plus that in the sponges in this study. More precise amount of blood loss should be evaluated by formulas based on laboratory data, such as Gross' equation. Fourth, larger sample size and more confounders should be used to explore the risk factors for hypothermia. Besides, we did not use active warming devices routinely. Forced air warming would be employed when the temperature dropped to 36°C. However, we did not collect the exact number of patients receiving forced air warming, which may affect the analysis.

In conclusion, intraoperative temperature and hypothermia were associated with intraoperative blood loss and perioperative transfusion rates in TKA. Intraoperative temperature was negatively related to the amount of blood loss, and hypothermia was the independent risk factor for transfusion in THA. Therefore, strategies to maintain normothermia should be employed to reduce blood loss and transfusion rates in these patients.

## Figures and Tables

**Table 1:** Patient's demographic and clinical characteristics.

Characteristics	TJA (n = 616)	TKA (n = 272)	THA (n = 344)
Age (years (mean ± SD))	65.9 ± 11.8	68.0 ± 7.3	64.2 ± 14.1
Gender			
Male (%)	190 (30.8)	50 (18.4)	140 (40.7)
Female (%)	426 (69.2)	222 (81.6)	204 (59.3)
BMI (kg/m^2^ (mean ± SD))	24.8 ± 3.9	26.2 ± 3.7	23.6 ± 3.6
Diabetes (%)	85 (13.8)	50 (18.4)	35 (10.2)
Hypertension (%)	237 (38.5)	122 (44.9)	115 (33.4)
Malignancy (%)	15 (2.4)	8 (2.9)	7 (2.0)
Diagnosis			
OA (%)	299 (48.5)	246 (90.4)	53 (15.4)
RA (%)	33 (5.4)	26 (9.6)	7 (2.0)
Femoral neck fracture (%)	147 (23.9)	—	147 (42.7)
ONFH (%)	79 (12.8)	—	79 (23.0)
DDH (%)	35 (5.7)	—	35 (10.2)
AS (%)	23 (3.7)	—	23 (6.7)
Operation time (min (mean ± SD))	111.9 ± 28.0	124.1 ± 23.4	102.2 ± 27.6
PT (s (mean ± SD))	11.5 ± 1.0	11.3 ± 0.9	11.6 ± 1.0
INR (mean ± SD)	1.01 ± 0.08	0.99 ± 0.08	1.01 ± 0.09
APTT (s (mean ± SD))	26.9 ± 4.5	26.0 ± 3.8	27.6 ± 4.9
Hb (g/l (mean ± SD))	127.7 ± 14.5	126.2 ± 13.4	129.0 ± 15.2
PLT (10^9^/l (mean ± SD))	203.2 ± 66.6	206.8 ± 69.2	200.3 ± 64.5
Intraoperative temperature (°C (mean ± SD))	36.4 ± 0.2	36.4 ± 0.2	36.3 ± 0.1
Hypothermia (%)	83 (13.5)	38 (14.0)	45 (13.1)
Intraoperative blood loss (ml (mean ± SD))	208.7 ± 131.3	196.0 ± 103.2	218.7 ± 149.3
Allogenic blood transfusion (%)	94 (15.3)	48 (17.6)	46 (13.4)

TJA: total joint arthroplasty; TKA: total knee arthroplasty; THA: total hip arthroplasty; SD: standard deviation; BMI: body mass index; OA: osteoarthritis; RA: rheumatoid arthritis; ONFH: osteonecrosis of the femur head; DDH: developmental dysplasia of the hip; AS: ankylosing spondylitis; PT: prothrombin time; INR: international normalized ratio; APTT: activated partial thromboplastin time; Hb: hemoglobin; PLT: platelet count.

**Table 2:** Univariate analysis of the risk factors for intraoperative hypothermia in TKA.

Variable	No hypothermia (n = 234)	Hypothermia (n = 38)	P value
Age (years (mean ± SD))	68.1 ± 7.2	66.9 ± 8.3	0.326
Female gender (%)	193 (82.5)	29 (76.3)	0.363
BMI (kg/m^2^ (mean ± SD))	26.3 ± 3.8	25.6 ± 3.4	0.268
Diabetes (%)	42 (18.0)	8 (21.1)	0.647
Hypertension (%)	108 (46.2)	14 (36.8)	0.284
Malignancy (%)	7 (3.0)	1 (2.6)	0.903
Diagnosis of RA (%)	21 (9.0)	5 (13.2)	0.416
Operation time (min (mean ± SD))	123.4 ± 23.4	128.8 ± 23.4	0.185
PT (s (mean ± SD))	11.4 ± 0.9	11.3 ± 1.1	0.501
INR (mean ± SD)	0.99 ± 0.08	0.99 ± 0.09	0.786
APTT (s (mean ± SD))	26.1 ± 3.9	25.5 ± 3.6	0.418
Hb (g/l (mean ± SD))	126.0 ± 13.1	126.9 ± 15.6	0.706
PLT (10^9^/l (mean ± SD))	207.9 ± 68.2	200.4 ± 75.6	0.535
Blood loss (ml (mean ± SD))	189.0 ± 99.3	239.5 ± 116.9	0.005∗
Transfusion (%)	33 (14.1)	15 (39.5)	<0.001∗

^∗^ *P* < 0.05 was considered statistically significant. SD: standard deviation; BMI: body mass index; RA: rheumatoid arthritis; PT: prothrombin time; INR: international normalized ratio; APTT: activated partial thromboplastin time; Hb: hemoglobin; PLT: platelet count.

**Table 3:** The association of intraoperative temperature and hypothermia with intraoperative blood loss and perioperative blood transfusion in TKA.

	Intraoperative blood loss		Perioperative blood transfusion	
	Unstandardized coefficients	P value	OR	P value
Intraoperative temperature	-62.700	0.045∗	0.080	0.006∗
Hypothermia	35.974	0.042∗	4.468	<0.001∗

^∗^ *P* < 0.05 was considered statistically significant. OR: odds ratio.

**Table 4:** Univariate analysis of the risk factors for intraoperative hypothermia in THA.

Variable	No hypothermia (n = 299)	Hypothermia (n = 45)	P value
Age (years (mean ± SD))	64.0 ± 14.3	65.9 ± 12.5	0.397
Female gender (%)	173 (57.9)	31 (68.9)	0.160
BMI (kg/m^2^ (mean ± SD))	23.5 ± 3.6	23.9 ± 3.6	0.539
Diabetes (%)	28 (9.4)	7 (15.6)	0.200
Hypertension (%)	95 (31.8)	20 (44.4)	0.093
Malignancy (%)	7 (2.3)	0	0.300
Diagnosis	—	—	0.377
Operation time (min (mean ± SD))	101.7 ± 27.4	105.6 ± 29.2	0.372
PT (s (mean ± SD))	11.6 ± 0.9	11.6 ± 1.2	0.890
INR (mean ± SD)	1.02 ± 0.08	1.01 ± 0.11	0.623
APTT (s (mean ± SD))	27.5 ± 4.8	27.9 ± 5.6	0.656
Hb (g/l (mean ± SD))	129.2 ± 15.3	128.0 ± 14.9	0.629
PLT (10^9^/l (mean ± SD))	201.1 ± 64.9	194.5 ± 62.2	0.522
Blood loss (ml (mean ± SD))	214.5 ± 137.3	246.7 ± 212.2	0.178
Transfusion (%)	35 (11.7)	11 (24.4)	0.019∗

^∗^ *P* < 0.05 was considered statistically significant. SD: standard deviation; BMI: body mass index; PT: prothrombin time; INR: international normalized ratio; APTT: activated partial thromboplastin time; Hb: hemoglobin; PLT: platelet count.

**Table 5:** The association of intraoperative temperature and hypothermia with intraoperative blood loss and perioperative blood transfusion in THA.

	Intraoperative blood loss		Perioperative blood transfusion	
	Unstandardized coefficients	P value	OR	P value
Intraoperative temperature	-153.067	0.002∗	0.122	0.073
Hypothermia	30.138	0.152	2.615	0.031∗

^∗^ *P* < 0.05 was considered statistically significant. OR: odds ratio.

## Data Availability

The data used to support the findings of this study are available from the corresponding author upon request.
